# LC3B is not recruited along with the autophagy elongation complex (ATG5-12/16L1) at HCV replication site and is dispensable for viral replication

**DOI:** 10.1371/journal.pone.0205189

**Published:** 2018-10-04

**Authors:** Ahmed M. Fahmy, Marwa Khabir, Matthieu Blanchet, Patrick Labonté

**Affiliations:** 1 INRS-Institut Armand-Frappier, Institut National de la Recherche Scientifique, Laval, Canada; 2 Molecular Medicine Research Group, Department of Reproductive Medicine, Division of Medical Research, National Research Centre (NRC), Dokki, Egypt; Scripps Research Institute, UNITED STATES

## Abstract

Hepatitis C virus (HCV) infection is known to induce autophagosome accumulation as observed by the typical punctate cytoplasmic distribution of LC3B-II in infected cells. Previously, we showed that viral RNA-dependent RNA polymerase (NS5B) interacts with ATG5, a major component of the autophagy elongation complex that is involved in the formation of double-membrane vesicles (DMV), and demonstrated that the autophagy elongation complex (ATG5-12/16L1) but not LC3B is required for proper membranous web formation. In this study, the colocalization and *in situ* interaction of all HCV replicase components with the constituent of the autophagy elongation complex and LC3B were analyzed. The results clearly show the recruitment of the elongation complex to the site of viral replication. Using *in situ* proximity ligation assay, we show that ATG5, but not ATG16L1, interacts with several HCV replicase components suggesting that the recruitment is directed via the ATG5-12 conjugate. Interestingly, no E3-like conjugation activity of ATG5-12/16L1 can be detected at the at HCV replication site since LC3B-II is not found along with the elongation complex at the site of viral replication. In agreement with this result, no sign of *in situ* interaction of LC3B with the replicase components is observed. Finally, using dominant negative forms of ATG proteins, we demonstrate that ATG5-12 conjugate, but not LC3-II formation, is critical for viral replication. Altogether, these findings suggest that although HCV needs the elongation complex for its replication, it has developed a mechanism to avoid canonical LC3-II accumulation at viral replication site.

## Introduction

Hepatitis C virus (HCV) infection leads to a wide spectrum of diseases ranging from asymptomatic to end-stage liver diseases including cirrhosis and hepatocellular carcinoma [1]. HCV is an enveloped, positive-strand RNA virus that belongs to the *Flaviviridae* family. The HCV genome is approximately 9.6 kb in length and consists of a single ORF flanked by two non-coding regions (NCRs). The translated polyprotein is processed by cellular and viral proteases into the structural proteins (core, E1, and E2) and the nonstructural proteins (p7, NS2, NS3, NS4A, NS4B, NS5A, and NS5B)[2]. HCV replication is marked by the formation of a membrane-associated replication complex with a unique membrane alteration referred to as the membranous web [3]. The majority of the membranous structures found within the HCV replication site are composed of double membrane vesicles (DMVs) suggesting that autophagy is involved in the establishment of the HCV replication scaffold [4–6]. DMVs are now strongly suspected of being the primary site of HCV replicase localization where active viral RNA replication occurs [4, 7, 8].

Autophagy is an intracellular catabolic process essential to maintain cell homeostasis which is particularly noticeable under nutrient-deprivation conditions such as starvation [9]. In addition, autophagy provides a cell-autonomous defense system against microbial infections and intracellular pathogens via the autophagosome/lysosome pathway [10, 11]. Autophagy is initiated by the formation of the isolation membrane, the phagophore, which extends to form a closed DMV known as the autophagosome. This structure then fuses with a lysosome to form an autolysosome. The fusion allows the degradation of the autophagosomal cargo by lysosomal enzymes.

Although autophagy has antiviral capability, several viruses and especially positive-strand RNA viruses can use the autophagy machinery for their own benefit [12–16]. Among them, HCV is known to induce accumulation of LC3B-II punctate structures [17, 18]. Furthermore, it was shown that at least a part of the autophagy process is absolutely required for the HCV life cycle *in vitro* [19, 20]. It has been proposed that HCV may induce autophagosome formation through the unfolded protein response (UPR)[21, 22]. However, others have suggested that autophagy is triggered independently of the UPR in HCV-infected cells [23]. NS4B expression has been shown to be sufficient to induce the accumulation of autophagosomes as seen by the redistribution of diffused LC3 (LC3B-I) to punctate structures (LC3B-II) in NS4B-transfected cells [24]. It has also been demonstrated that induction of autophagy by HCV is important for the suppression of the antiviral interferon response [22, 25]. In addition to this indirect action of autophagy that favors the establishment and the maintenance of HCV, it has been suggested that autophagic proteins promote HCV replication by either facilitating protein translation [18] or virus maturation [20]. It was also shown that upon HCV infection, NS5A transcriptionally upregulates Beclin1, enhances phospho-mTOR expression, and thus, activates mTOR signaling pathway [26]. On the other hand, a more recent study proposed that HCV-induced autophagy occurs via inhibition of AKT-TSC-mTOR via ER stress [27]. In a previous study, we have shown that HCV RNA-dependent RNA polymerase (RdRp), the NS5B, colocalizes and interacts with ATG5, a component of the autophagy elongation complex and a key factor for the formation of autophagosomes [28]. We also showed that the autophagy elongation complex (ATG5-12/16L1) can be co-purified with the HCV membranous web and that its expression is essential for proper membranous web formation [29]. Therefore, we proposed that the ATG5-12/16L1 complex provide assistance in the formation of membranous structures used by the virus for its replication. The ATG5-12 is an E3-like conjugation enzymes required for LC3-II formation and incorporation on DMVs [30], which is essential for canonical autophagosome maturation. Therefore, it would be expected that LC3-II be localized at the HCV replication site along with the elongation complex. Importantly, recent studies demonstrate that incorporation of LC3-II within the replication complex of positives-sense RNA viruses triggers robust IFN-inducible antiviral responses that disrupt the viral replication shelter [31, 32]. In this study, we confirm that although the autophagy elongation complex is recruited at HCV replication site where it interacts with HCV replicase components as a proviral factor, LC3B-II does not interact *in situ* with HCV replicase and is not found at the HCV replication site suggesting that HCV has evolved strategies to avoid LC3 integration within its replication complex.

## Materials and methods

### Cell culture and reagents

Huh7 cells were obtained from Dr Ralf Bartenschlager and were cultured in Dulbecco’s modified Eagle’s Medium (DMEM) (Gibco), supplemented with 10% v/v fetal bovine serum (FBS) (Multicell), 100 U/ml penicillin, 100μg/ml streptomycin, 2 mM L-glutamine (Gibco) at 37°C, 5% CO2, in a humidified incubator.

### Plasmids and antibodies

hATG5 and hATG16L1 sequences were cloned into peGFP-C1 plasmid (Clontech) to form pGFP-ATG5 and pGFP-ATG16L1, respectively. The peGFP-LC3 construct was kindly provided by Dr. Tamotsu Yoshimori (Japan) [33]. The pmStrawberry-ATG4BC74A (ATG4B-DN), pcDNA3-mRuby2, and pCI-neo-hApg5-K130R-HA (ATG5-DN) constructs were purchased from Addgene (Cambridge, USA). The Flag-tagged ATG12 (pATG12) and its dominant-negative derivative pATG12ΔG140 (ATG12-DN) constructs were kindly provided by Dr. Adi Kimchi (Israel) [34]. Rabbit polyclonal anti-LC3, rabbit polyclonal anti-ATG5 (used for western blot), mouse monoclonal anti-Flag, and mouse monoclonal anti-β-actin antibodies were purchased from Sigma Aldrich (USA). Mouse monoclonal anti-ATG5 (used for immunofluorescence) and anti-P62 antibodies were purchased from Abnova (Taiwan). Rabbit polyclonal anti-ATG12 was purchased from Cell Signaling (USA). Mouse monoclonal anti-LC3 and rabbit polyclonal anti-ATG16L1 antibody were purchased from MBL (USA). Mouse monoclonal anti-dsRNA was purchased from English & Scientific Consulting (Hungary). Mouse monoclonal anti-HA was purchased from Roche (USA). Mouse monoclonal anti-Core was purchased from Virostat (USA). Mouse monoclonal anti-NS3 and anti-NS5A antibodies were purchased from BioFront (USA). Rabbit polyclonal anti-NS3 and NS5A were obtained from Dr. Olivier Nicolas. Rabbit polyclonal anti-NS4B and anti-NS5B antibodies were kindly provided by Drs. Kouacou Konan (USA) and Takaji Wakita (Japan), respectively. Mouse monoclonal anti- β-actin was purchased from Sigma Aldrich (USA).

### Preparation of viral stock and infections

The cell culture-derived HCV (HCVcc) JFH1 virus was generated in Huh7 cells by transfection of *in vitro-*transcribed full-length JFH1 RNA (MEGAscript, Ambion) and viral stocks were produced by infection of Huh7 cells at a multiplicity of infection (MOI) of 0.01, as described previously [35]. To reach 90% infected cells, huh7 cells were infected at MOI of 0.01 and passaged for 7 days then analyzed by immunofluorescence using anti-NS5A antibody.

### Western blot analysis

Cells were lysed in 300 μl of lysis buffer [25 mM Tris-HCl, 150 mM NaCl, 1 mM EDTA, 1% NP40, Complete protease inhibitor (Roche)]. Lysates were normalized for total protein content using the BCA protein assay kit (Pierce). Proteins were then resolved by SDS-PAGE, transferred to polyvinylidene fluoride (PVDF) membranes (Bio-Rad), blocked for 30 min at room temperature (RT) with PBS-5% milk, and then incubated overnight at 4°C with primary antibody in PBS-5% milk. After washing with 0.1% Tween 20 in PBS (PBST), membranes were incubated 1 h at RT with a goat-anti-rabbit or goat-anti-mouse IgG conjugated to horseradish peroxidase in PBS-5% milk. Protein bands were visualized with either the Super Signal West-Pico or -Femto chemiluminescence substrates (Pierce).

### Indirect immunofluorescence

Huh7 cells infected at greater than 90% were transfected with different plasmids as indicated in figure legends. At 24 h post-transfection, cells were trypsinized and grown on glass coverslips for another 24 h. The coverslips were then fixed with 4% formaldehyde in PBS for 10 min, washed in PBS and incubated in blocking buffer (PBS, 3% bovine serum albumin, 10% FBS, 0.1% Triton X-100) for 30 min at RT. For the detection of GFP-LC3, cells were permeabilized with 0.05% saponin to remove dispersed LC3 (LC3-I) [27]. After washing with PBS, the coverslips were incubated with primary antibodies in blocking buffer for 1 h at RT. Coverslips were then washed in PBS and incubated with either Alexa fluor-(488 or 568) goat anti-mouse IgG or Alexa fluor-(488 or 568) goat anti-rabbit IgG (Invitrogen) for 1h at RT. After washing, coverslips were mounted on glass slides with Prolong Antifade (Invitrogen) and examined with either a laser scanning confocal BioRad Radiance 2000 or a Zeiss LSM 780. The Manders’coefficient of colocalization was obtained using ImageJ software (NIH) in randomly selected regions that were positive for the targeted proteins from different cells. Manders’coefficient values over 0.4 were considered as strong colocalization.

### In situ proximity ligation assay (PLA)

Huh7 cells infected at greater than 90% were grown on glass coverslips for 24 h prior to the fixation with 4% formaldehyde in PBS for 10 min. They were then washed in PBS and incubated in blocking buffer (PBS, 3% bovine serum albumin, 10% FBS, 0.1% Triton X-100) for 30 min at RT. Coverslips were incubated with primary antibodies for 1 h at RT then washed three times with 1x wash buffer A (Duolink (and incubated with PLA probes (anti-rabbit plus and anti-mouse minus) diluted with the provided buffer in a humidity chamber for 1 h at 37°C. Coverslips were then washed three times with 1x wash buffer A and incubated with the ligation-ligase reaction solution in a humidity chamber for 30 min at 37°C. Amplification and mounting steps were performed according to manufacturer’s instructions. Mounted coverslips were examined with a laser scanning confocal microscope (Zeiss LSM 780). Each detected signal represents an interaction event. The analysis of PLA signal frequency was done using Duolink Image Tool.

### Quantification of HCV RNA by qRT-PCR

RNA was reverse transcribed with M-MLV (Invitrogen). Generated cDNA was used for qPCR (Taqman) as described earlier [36]. Results were analyzed using the comparative ΔCt method.

### Flow cytometry

Huh7 cells infected at greater than 90% were transfected with either mock (pcDNA3-mRuby2) or ATG4B-DN. At day 2 cells were trypsinized and washed twice with PBS. Dead cells were labeled using FVD780 (eBioscience). Cells were fixed with 4% formaldehyde for 10 min at RT, incubated with blocking buffer (PBS, 2% BSA, 0.2% Saponin) for 20 min at 4°C and incubated with primary antibodies diluted in blocking buffer for 30 min at 4°C. After washing with PBS, cells were incubated with Alexa fluor-488 goat anti-rabbit IgG (Invitrogen) for 30 min at 4°C. Cells were analyzed using BD LSRFortessa cell analyzer (BD Biosciences) and Cyflogic software (CyFlo Ltd).

### Statistical analyses

Results shown represents the means of at least three independent experiments. Either Student’s t-test or ANOVA analysis was performed to identify statistically significant differences. P values below 0.05 were considered statistically significant.

## Results

### Formation of the ATG5-12 conjugate and the autophagy elongation complex ATG5-12/16L1 in Huh7 cells

**Fig 1:**
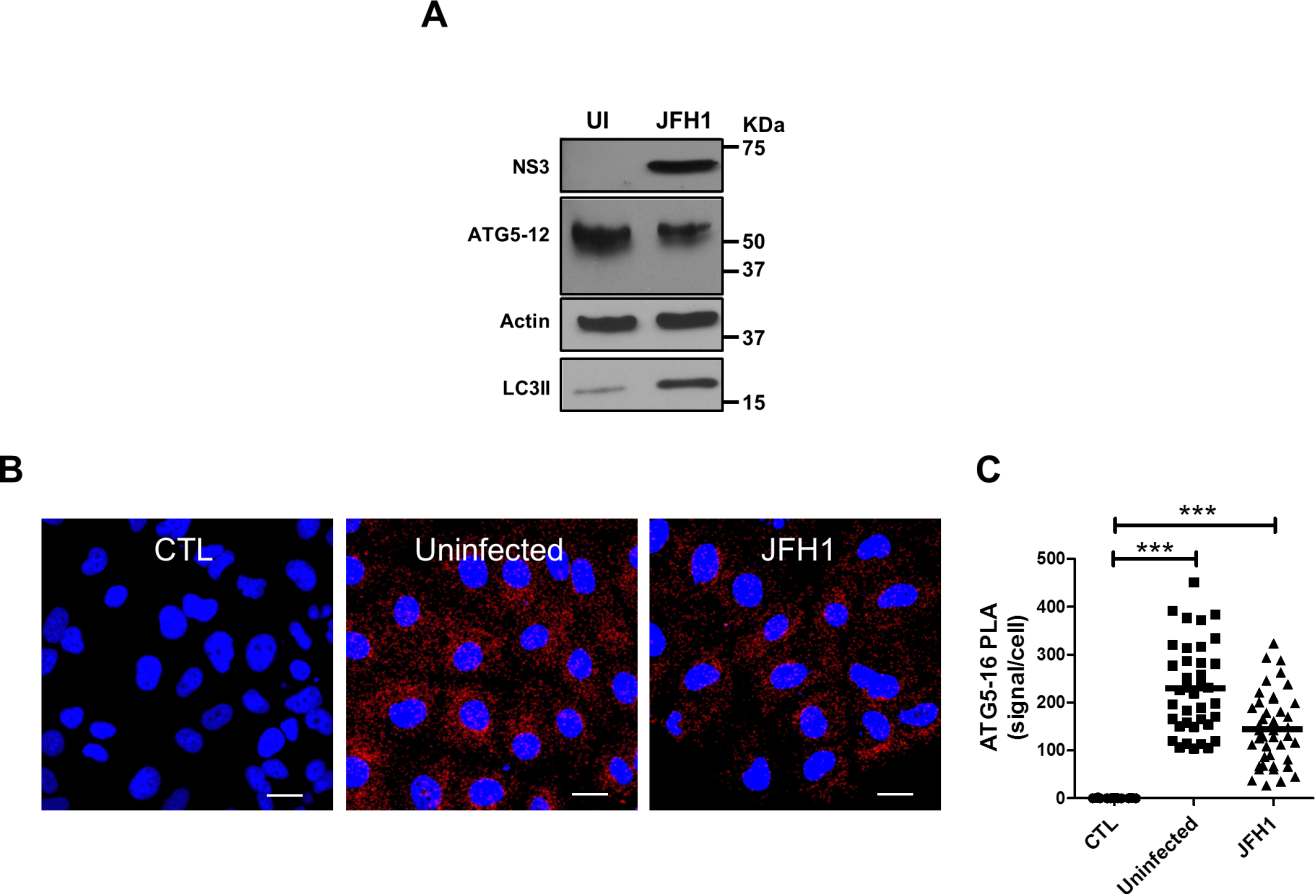
Formation of the autophagy elongation complex in Huh7 cells. A. Detection of the ATG5-12 conjugate by Western blot in mock (UI) and JFH1 infected Huh7 cells at more than 90% using an anti-ATG5 antibody. HCV infection and autophagosome accumulation were detected using anti-NS3 and anti-LC3 antibodies, respectively. β-actin represents loading control. B. *In situ* ATG5-12/16L1 complex formation was analyzed using PLA in JFH1-infected at more than 90% or uninfected cells. Cells were labeled for ATG5-12 and ATG16L1 using anti-ATG5 and anti-ATG16L1 respectively. CTL represents negative control lacking anti-ATG5 antibody. Nuclei were counterstained with DAPI (blue). Scale bar, 20μm. C. The frequency of PLA signals were significantly higher in both JFH1-infected and uninfected cells compared to control cells (CTL) (based on the count in 40 cells for each condition) (P<0.0001, 1way ANOVA).

We have shown in a previous study that HCV polymerase interacts with ATG5, a protein that participates in early events during induction of autophagy [28]. Since ATG5 is normally conjugated to ATG12, we first compared the conjugation status of ATG5 in Huh7 infected and uninfected cells. The results showed that the unconjugated form of ATG5 (32 kDa) was undetectable in both infected and uninfected cells. Indeed, ATG5 was exclusively detected as ATG5-12-conjugated form (55 kDa) (Fig 1A). The absence of detectable unconjugated ATG5 suggests that most of the ATG5 is readily conjugated to ATG12 in Huh7 cells, as previously reported for other cell types [37]. Additionally, HCV infection did not hamper this conjugation. Moreover, results show that infection induces the accumulation of LC3-II, a hallmark of induced autophagy (Fig 1A).

Once ATG5 is conjugated to ATG12, it can form a multimeric complex by association with ATG16L1 [38]. To assess whether this interaction occurs during infection, we investigated the presence of ATG5-12/16L1 complex by *in situ* proximity ligation assay (PLA). This novel technique has been used in several studies to detect specific *in situ* interactions [39–42]. Clearly, endogenous ATG5-12/16L1 complexes were detected in both infected and uninfected cells (Fig 1B and 1C). It is noteworthy that the count of interaction signals was slightly lower in infected cells. Although the significance of this reduction is not ascertained at the moment, our results suggest that HCV infection still allows the formation of the high molecular weight complex ATG5-12/16L1 that occurs spontaneously *in vitro* [43, 44].

### The ATG5-12 conjugate colocalizes and interacts with viral nonstructural proteins

**Fig 2:**
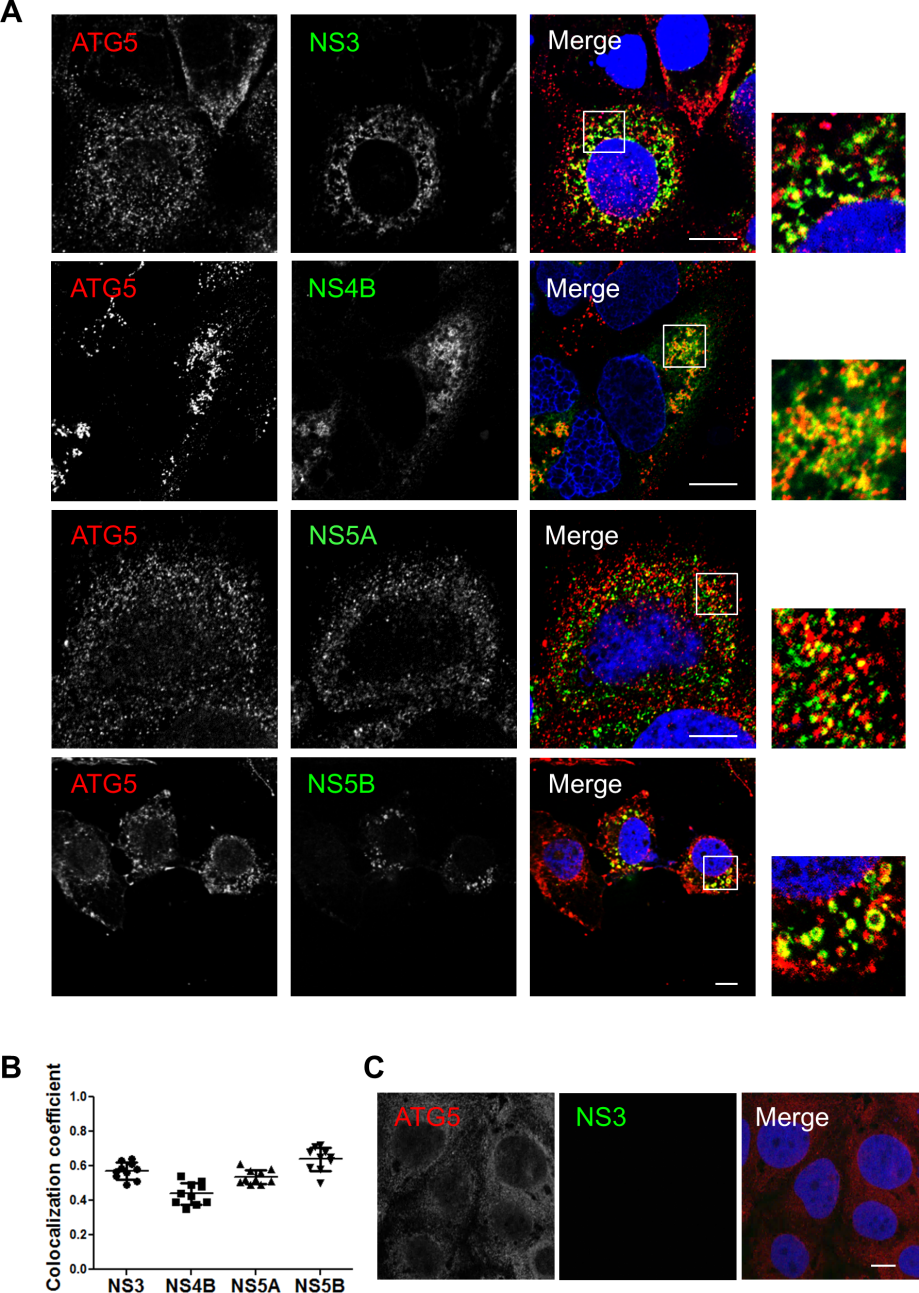
Components of the HCV replicase colocalize with ATG5-12 conjugate in Huh7 cells. A. JFH1-infected Huh7 cells at more than 90% were probed for endogenous ATG5-12 conjugate using a mouse anti-ATG5 antibody and HCV nonstructural proteins (NS3, NS4B, NS5A, and NS5B) using rabbit specific antibodies as described in the materials and methods section. The nuclei were stained with DRAQ5 (blue). Confocal microscopy images displaying subcellular localization of endogenous ATG5-12 conjugate and viral NS3, NS4A, NS5A, and NS5B in merged image panels are shown. Marked colocalization between endogenous ATG5-12 conjugate and components of the viral replicase (NS3, NS5A, and NS5B) or the membranous web (NS4B) was observed. Scale bar, 10μm. B. The average of colocalized pixels of ATG5-12 and HCV nonstructural proteins (n = 5 cells, 10 arbitrary positions) was determined. The values of overlapping fluorescence signal with HCV nonstructural proteins were calculated using Manders’ colocalization coefficient. C. Localization of ATG5-12 in uninfected cells. Uninfected Huh7 cells were immunostained for ATG5-12 using a mouse anti-ATG5 antibody. Scale bar, 10μm.

**Fig 3:**
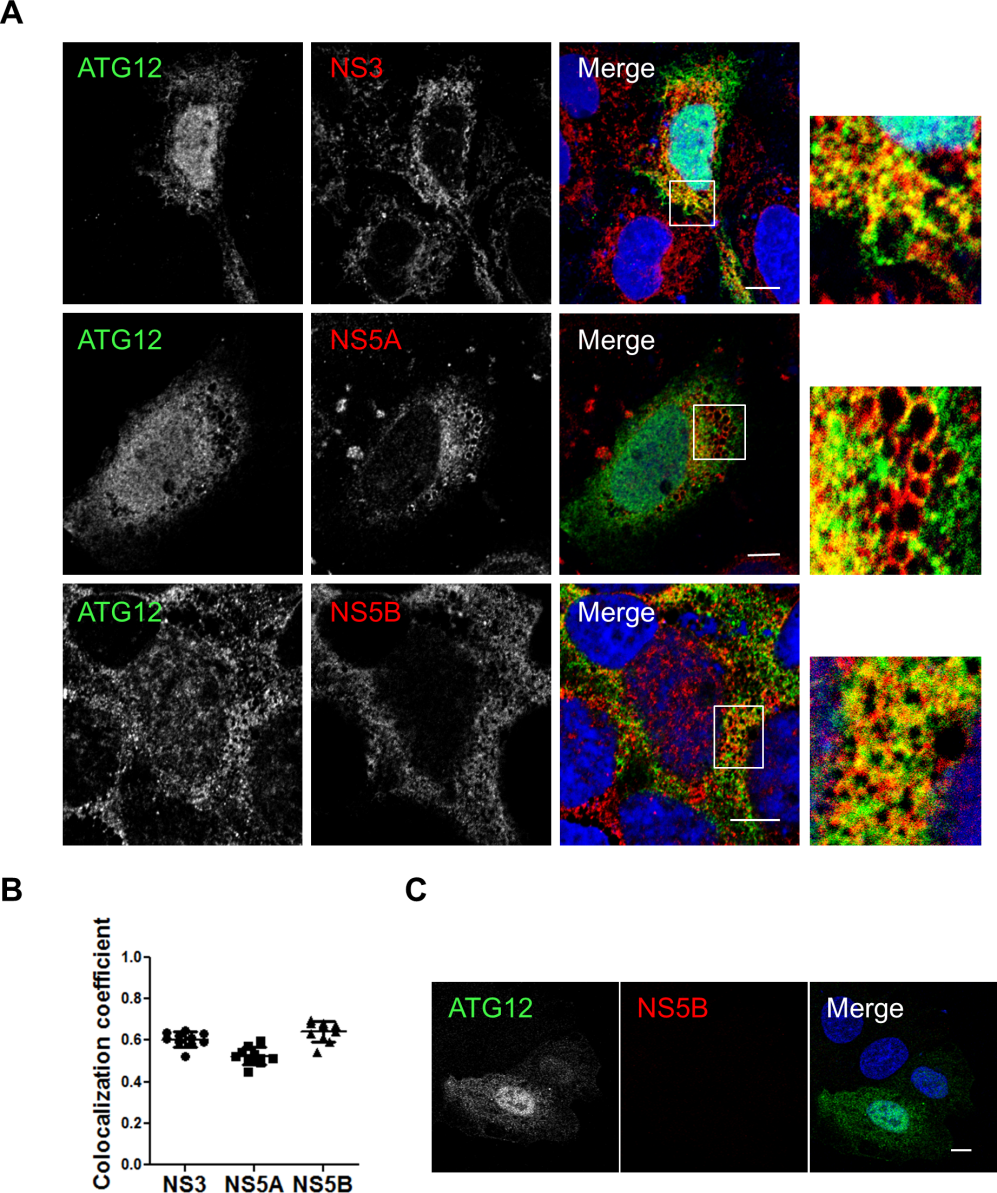
HCV nonstructural proteins colocalize with ATG12 protein in Huh7 cells. A. Huh7 cells were infected with HCVcc and then transfected with recombinant Flag-tagged ATG12. Confocal microscopy images displaying subcellular localization of ATG12 (green) and components of the viral replicase (NS3, NS5A and NS5B) (red) in merged images are shown. Scale bar, 10μm. B. The average colocalization of ATG12 and HCV nonstructural proteins (n = 5 cells, 10 arbitrary positions) was calculated using Manders’ colocalization coefficient. C. Localization of flag-tagged ATG12 in uninfected cells. Uninfected Huh7 cells were immunostained for ATG12 using a mouse anti-flag antibody. Scale bar, 10μm.

**Fig 4:**
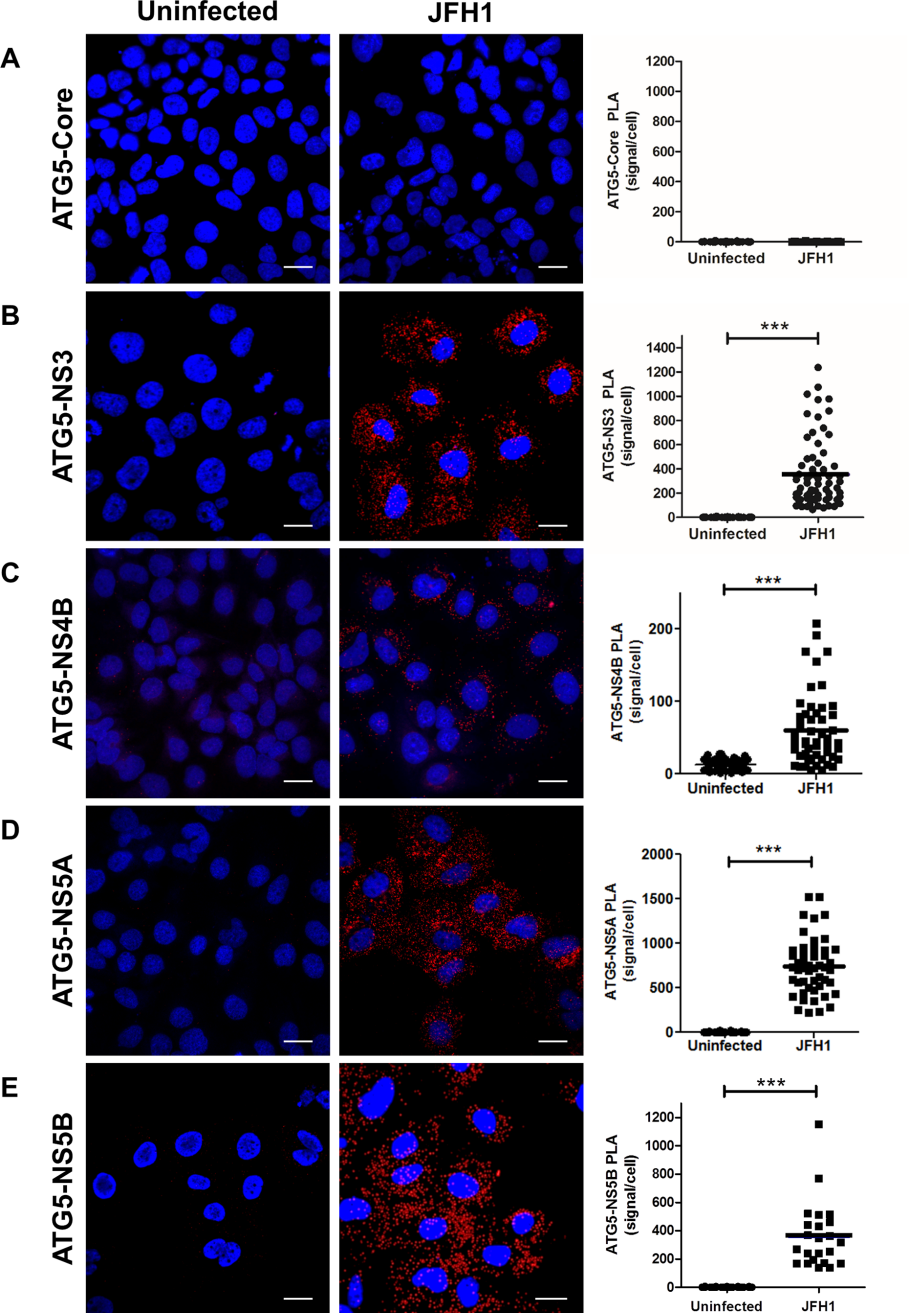
Assessment of ATG5-12 interactions with viral proteins in infected cells as observed by proximity-ligation assay (PLA). JFH1-infected at more than 90% or uninfected cells were fixed and processed for detection of ATG5-12-Core, ATG5-12-NS3, ATG5-12-NS4B, ATG5-12-NS5A or ATG5-12-NS5B complexes by PLA using appropriate antibodies. Nuclei were stained with DAPI (blue). Each PLA signal (red dot) indicates one interaction and were calculated as described in materials and methods section. In A, no significant difference in the frequency of PLA signals between JFH1-infected (n = 50) cells compared to uninfected controls (n = 50) indicating undetectable interaction between ATG5-12 conjugate and core. However, the incidence of PLA signals in B-E was significantly higher in JFH1-infected (n ≥23) cells compared to uninfected negative controls (N = 50) (*P*<0.0001, Student’s t-test) indicating complexes formation between ATG5-12 and NS3, NS4B, NS5A and NS5B. Scale bar, 20μm.

We then assessed the colocalization between the endogenous ATG5-12 conjugate and the components of the viral replicase in JFH1-infected Huh7 cells using an anti-ATG5 antibody. The results presented in Fig 2A and 2B show distinct membrane-like structures that are positive for the ATG5-12 conjugate as well as for HCV NS3, NS4B, NS5A, and NS5B. The distribution of ATG5-12 in uninfected cells is shown in Fig 2C. We also confirmed the colocalization of the ATG5-12 conjugate with the nonstructural viral proteins using an ATG12-Flag protein (Fig 3). These results support our previous study showing a crucial role of ATG5-12 in membranous web formation. Indeed, the high level of colocalization obtained suggests a putative direct interaction. Therefore PLA was performed to evaluate the *in situ* interaction between the ATG5-12 conjugate and components of the viral replicase (Fig 4). As a positive control, we started by analyzing the known ATG5/NS5B interaction in infected cells. The result showed that on average, 380 ATG5-12/NS5B interaction signals were detected per infected cells (Fig 4E). We then sought to screen for all other possible interactions of ATG5-12 conjugate and HCV non-structural proteins for which colocalizations were observed. As we showed earlier, NS3, NS4B and NS5A colocalize with ATG5-12 (Figs 2 and 3). Indeed, PLA experiments revealed that ATG5 interacts with these viral proteins *in situ* (Fig 4B–4D). In contrast, no interaction was observed between endogenous ATG5 and the viral core protein (Fig 4A). Since no colocalization was detectable in infected cells between these two proteins (data not shown), this PLA experiment was used as a specificity control of the assay. The PLA results, along with the colocalization findings from Figs 2 and 3, strengthen the hypothesis of the involvement of ATG5-12 conjugate in HCV replication.

### The autophagy elongation complex (ATG5-12/ATG16L1) is found at the HCV replication site

**Fig 5:**
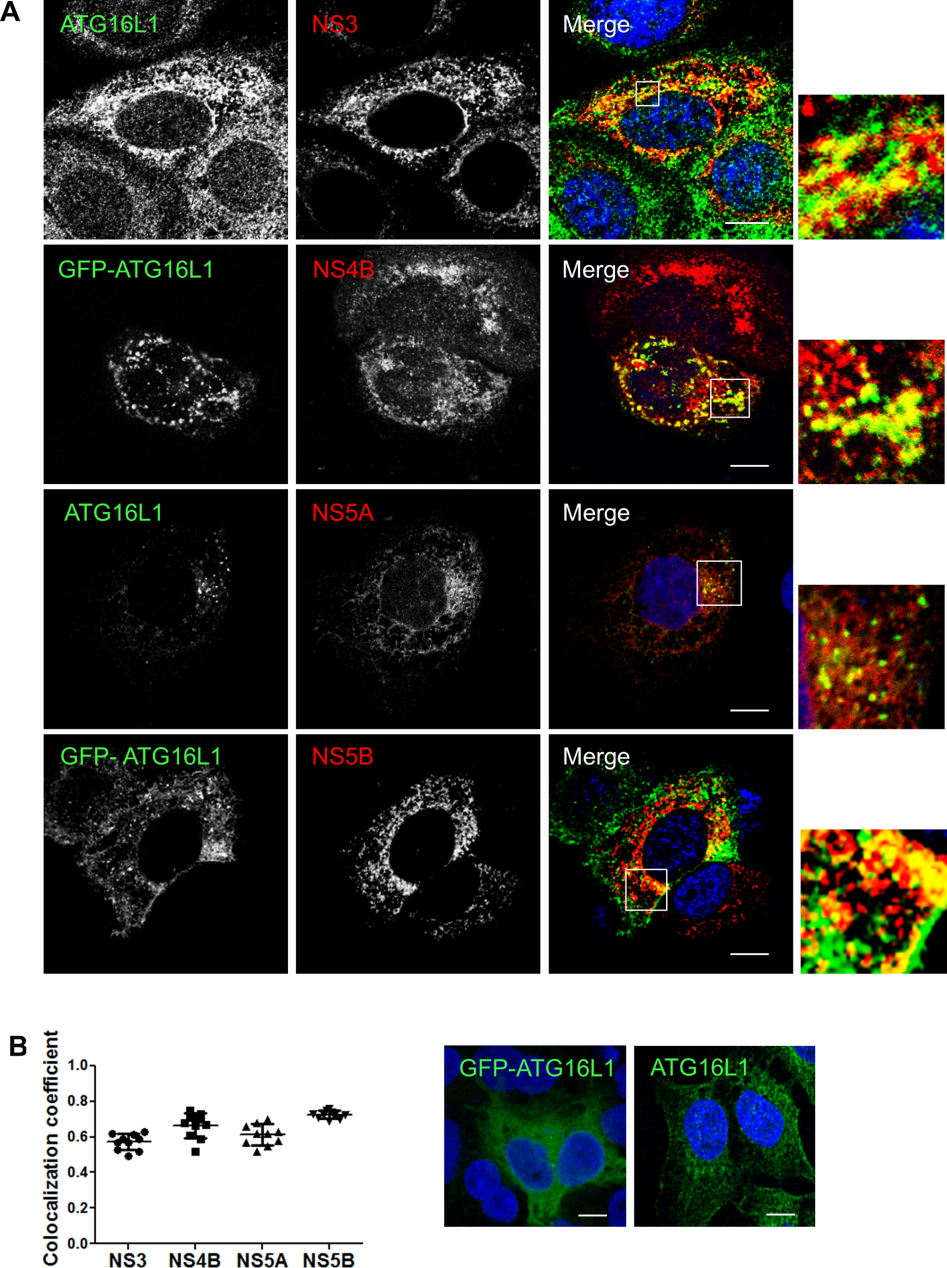
HCV nonstructural proteins colocalize with ATG16L1 in Huh7 cells. A. Huh7 cells infected with JFH1 at more than 90% and then transfected with pGFP-ATG16L1 or immunostained for endogenous ATG16L1 using a rabbit specific antibody. Confocal microscopy images displaying subcellular localization of GFP-ATG16L1 or endogenous ATG16L1 and viral NS3, NS4B, NS5A, and NS5B are shown. Scale bar, 10μm. B. The values of overlapping fluorescence signal of ATG16L1 and GFP-ATG16L1 with HCV nonstructural proteins were calculated using Manders’ colocalization coefficient (n = 5 cells, 10 arbitrary positions). C. Uninfected Huh7 cells transfected with pGFP-ATG16L1 (left) or immunostained for endogenous ATG16L1 (right) are depicted. ATG16L1 is shown in green and the nucleus in blue (Dapi). Scale bar, 10μm.

**Fig 6:**
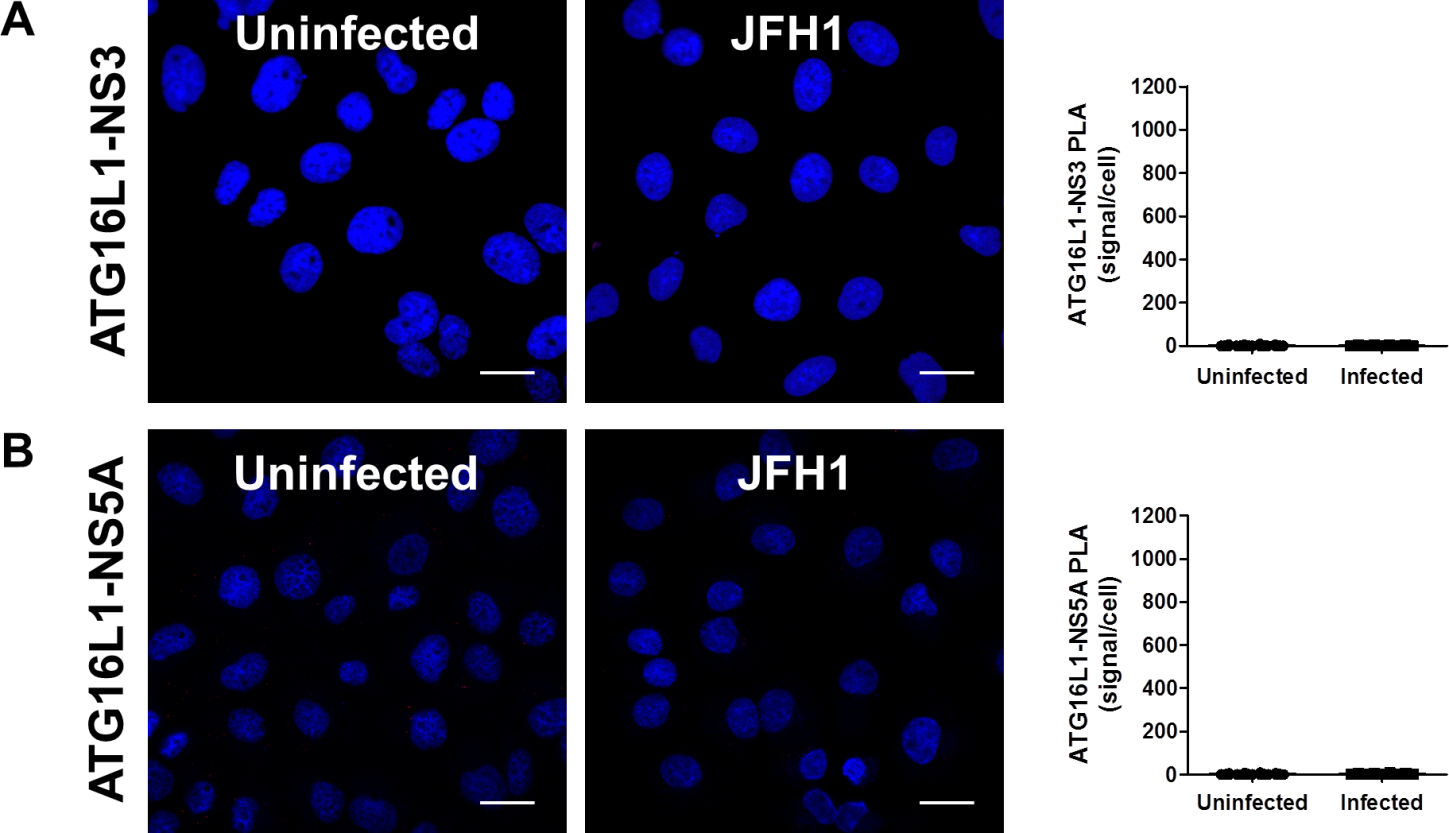
Interaction of the autophagy elongation complex with HCV non-structural proteins is not via ATG16L1. Using proximity-ligation assay (PLA), no *in situ* interaction could be observed between ATG16L1 and NS3 (A) or NS5A (B) as indicated by the frequency of PLA signals between JFH1-infected (n = 50) cells compared to uninfected controls (n = 50). Scale bar, 20μm.

In order to complete its normal functions, the ATG5-12 conjugate associates with ATG16L1 to form the autophagy elongation complex that allows the expansion of the autophagosomal membrane [38, 45]. Therefore, we sought to determine if the elongation complex, and not only ATG5-12 conjugate, is recruited to the site of viral replication. The subcellular localization of either the endogenous ATG16L1 or a GFP-tagged human ATG16L1 protein was monitored in infected Huh7 cells. The GFP-ATG16L1 was used only when detection of endogenous ATG16L1 was not readily possible due to conflict in antibody species. Results show marked localization between ATG16L1 and several HCV nonstructural proteins that constitute the viral replicase as well as the NS4B protein (Fig 5). Despite the obvious colocalization of ATG16L1 with NS3 and NS5A, we were unable to detect *in situ* interaction of endogenous ATG16L1 and these proteins in infected cells using PLA (Fig 6A and 6B). These results suggest that the interaction of ATG5-12/16L1 with viral NS3 and NS5A occurs through ATG5-12 rather than ATG16L1.

**Fig 7:**
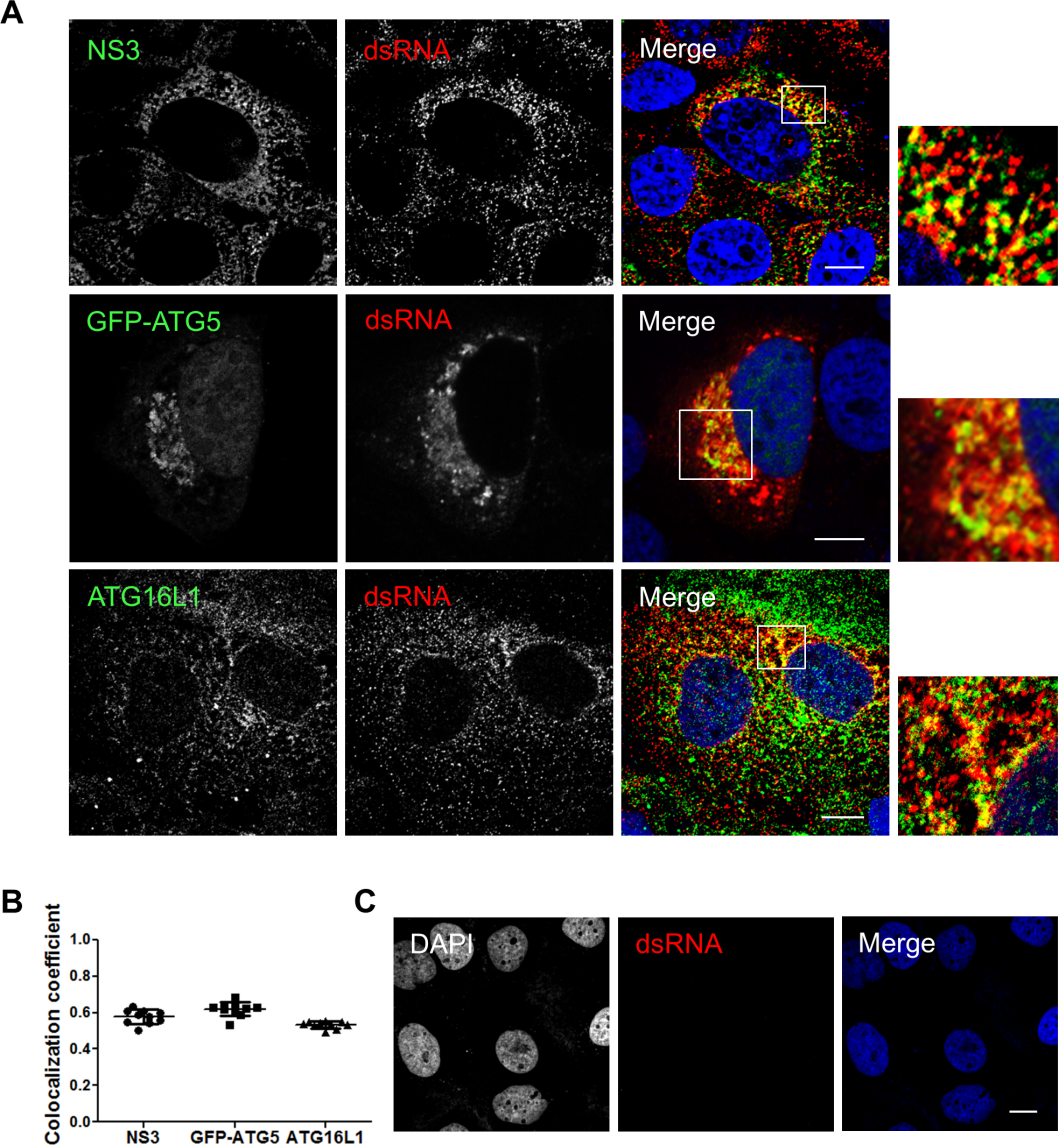
The autophagy elongation complex colocalizes with HCV replicative intermediate dsRNA. A. JFH1-infected Huh7 cells at more than 90% were immunostained for dsRNA and NS3 or ATG16L1. Alternatively, infected cells were transfected with pGFP-ATG5 and analyzed by confocal microscopy for dsRNA and GFP-ATG5. Scale bar, 10μm. B. The average of percent colocalization of NS3, GFP-ATG5 or ATG16L1 with dsRNA was determined. The values of overlapping fluorescence signals with dsRNA proteins were calculated using Manders’ colocalization coefficient (n = 5 cells, 10 arbitrary positions). C. Uninfected Huh7 cells immunostained for dsRNA. Negative staining shows specificity of dsRNA utilized in this experiment. Scale bar, 10μm.

Previously, we demonstrated that the elongation complex is found within the membranous web and is essential for its formation [29]. To support the hypothesis of an *in situ* interaction observed between ATG5-12/16L1 and HCV replicase components taking place, at least in part, within the replication complex, we analyzed the colocalization of the elongation complex with dsRNA (Fig 7). In infected cells, most of the dsRNA is expected to represent the HCV replication intermediate and thus, the replication site. As a positive control, the dsRNA-NS3 colocalization was assessed. HCV dsRNA markedly colocalized with the viral NS3 protein which harbors helicase activity and is known to be a constituent of the replicase (Fig 7A and 7B). The specificity of the dsRNA antibody was confirmed in uninfected Huh7 cells (Fig 7C). These results show that HCV dsRNA colocalizes with both ATG5-12 conjugate and ATG16L1 (Fig 7A and 7B), thus confirming the presence of ATG5-12 and ATG16L1 at the HCV replication site.

### LC3B-II is not recruited at the replication site and is not essential for HCV replication

**Fig 8:**
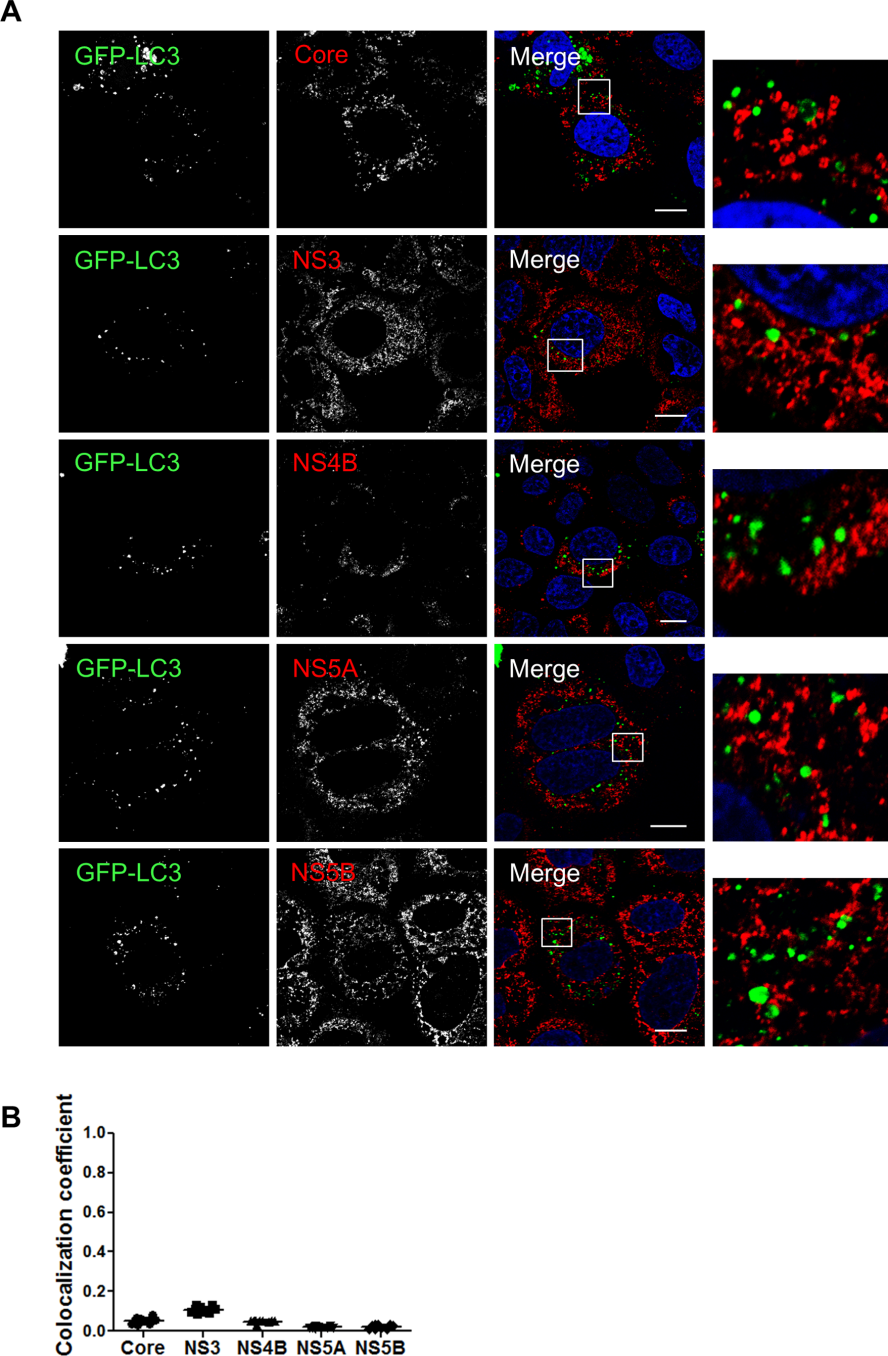
LC3 does not colocalize with HCV proteins. A. Huh7 cells were infected with JFH1 and then transfected with GFP-LC3. Confocal microscopy images displaying subcellular localization of GFP-LC3 and HCV core, NS3, NS4B, NS5A and NS5B are presented. Scale bar, 10μm. B. The values of overlapping fluorescence signal of GFP-LC3 with HCV nonstructural proteins were calculated using Manders’ colocalization coefficient (n = 5 cells, 10 arbitrary positions).

**Fig 9:**
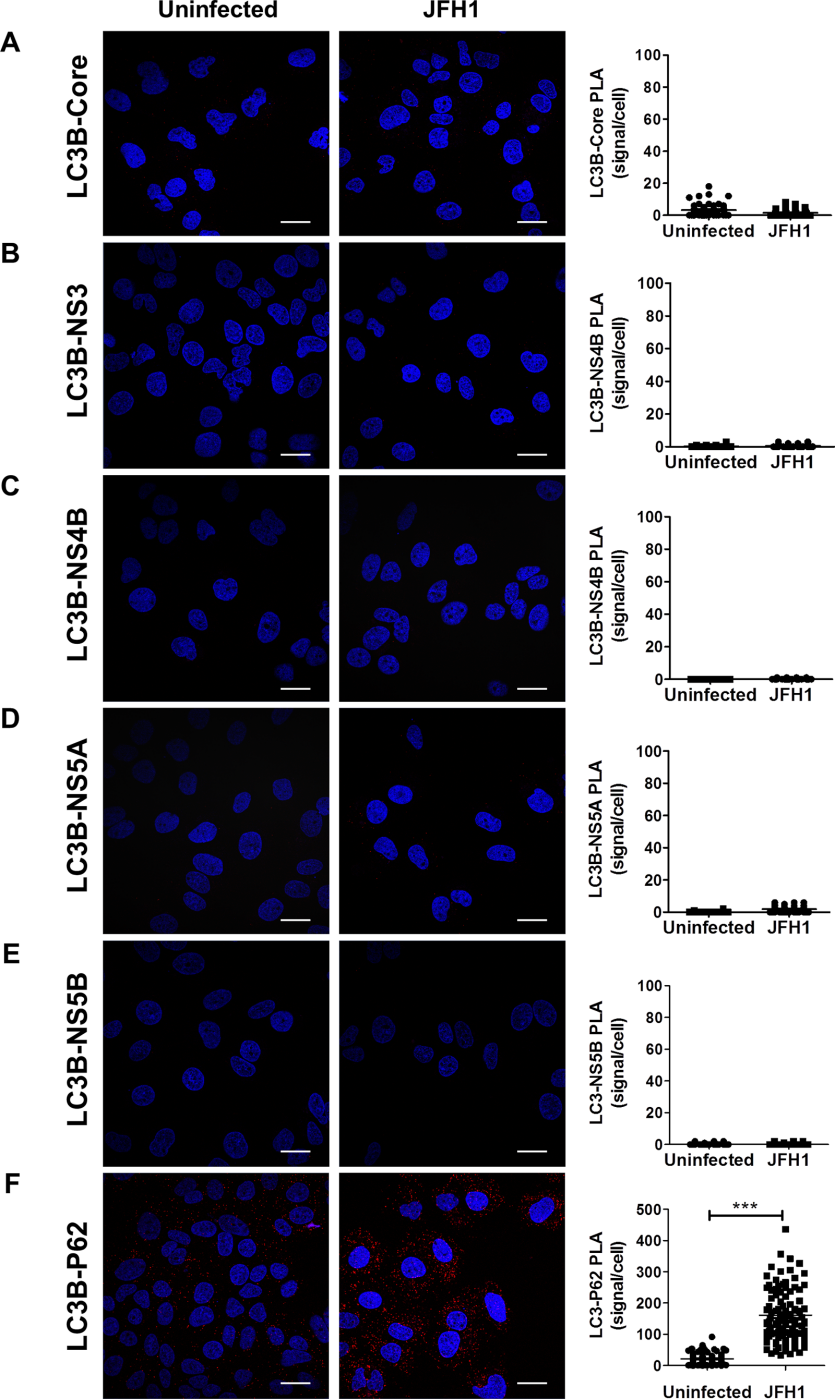
Assessment of LC3 interactions with viral proteins in infected cells as observed by proximity-ligation assay (PLA). JFH1-infected at more than 90% or uninfected cells were fixed and processed for detection of LC3-Core, LC3-NS3, LC3-NS4B, LC3-NS5A, LC3-NS5B or LC3-P62 (positive control) complexes by PLA using appropriate antibodies. Nuclei were stained with DAPI (blue Interaction signals were counted as described in materials and methods. In A-E, no significant difference in the frequency of PLA signals between JFH1-infected (n = 50) cells compared to uninfected controls (n = 50) indicating undetectable interaction between LC3 and the viral proteins. F. As control, the *in situ* interaction between LC3 and its natural ligand P62 was measured in both infected and uninfected cells. Interestingly, the incidence of PLA signals in F was significantly higher in JFH1-infected cells compared to uninfected negative controls (N = 50) (*P*<0.0001, Student’s t-test). Scale bar, 20μm.

As mentioned earlier, the presence of the E3-like conjugation enzyme ATG5-12/16L1 at HCV replication site should normally result in the addition of phosphatidylethanolamine (PE) to LC3-I allowing its membrane incorporation, as LC3-II, at this site. In a recent study, using knockdown experiments, we showed that ATG5-12, but not LC3, is important for proper membranous web formation [29]. With that in mind, we wondered if LC3-II is actually incorporated at HCV replication site. Because all members of the HCV replicase are known to be membrane associated, incorporation of LC3-II at the replication site can be evaluated by its colocalization with HCV proteins that reside within the replication site. Interestingly, despite strong colocalization of the elongation complex with HCV non-structural proteins (Figs 2, 3 and 5), no colocalization signals were observed between GFP-LC3II and HCV proteins (Fig 8). In agreement with this result, we were unable to detect any *in situ* interaction between endogenous LC3 and HCV proteins (Fig 9). As control, the known association of LC3 with its receptor P62 was compared in HCV infected and uninfected cells. As expected, the interaction was detected in either cases but was clearly more abundant in HCV infected cells (Fig 9F). This result is in favor of a model where HCV-induced autophagosome accumulation with a concomitant blockage of the autophagic flux as previously suggested [21, 46]. Altogether, these results demonstrate that LC3-II cannot be detected at the HCV replication site. We cannot exclude that LC3-II is transiently incorporated in membrane at the replication site and rapidly removed and/or degraded. However, clearly, LC3-II does not accumulate at the HCV replication site which implies that HCV has evolved to block canonical progression of autophagy at this site.

**Fig 10:**
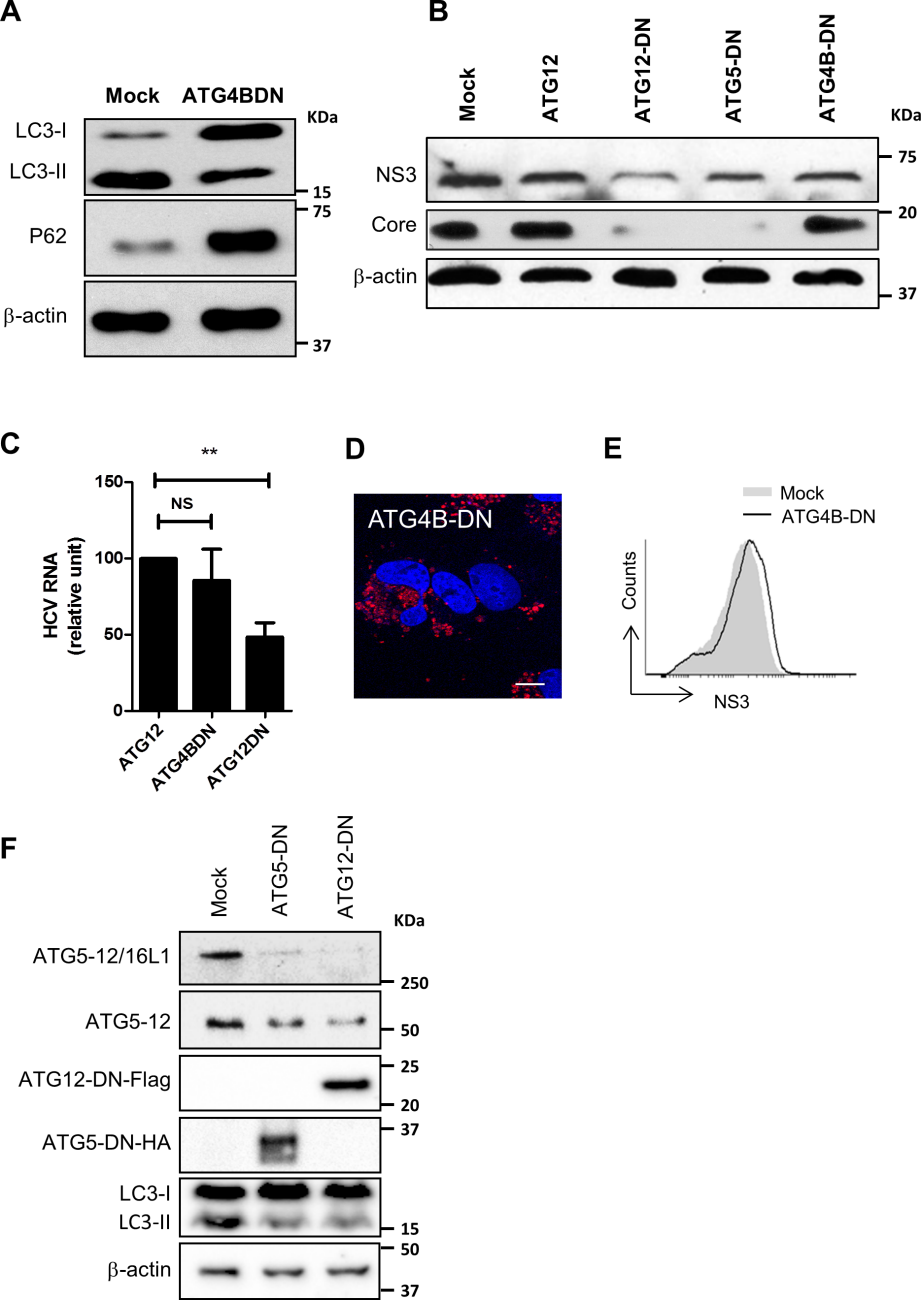
ATG5-12 conjugation but not LC3-II formation is important for the HCV lifecycle. A. Huh7 cells were either transfected with an empty plasmid (mock) or a plasmid encoding an enzymatically inactive dominant negative form of ATG4B (ATG4B-DN). Cell lysates were analyzed by Western blot at 72 h post-transfection for LC3-I to LC3-II conversion and P62 accumulation. B. JFH1-infected Huh7 cells (>90% infected) were transfected with plasmids encoding ATG12 or the dominant negative forms of ATG5, ATG12, or ATG4B. Cell lysates of transfected cells were analyzed 72 h post-transfection for HCV core and helicase expression using anti-NS3 by western blot. β-actin was used for normalization. C. Infected Huh7 cells (>90% infected) were transfected with a plasmid encoding ATG12, ATG4B-DN or ATG12DN. Cell lysates of transfected cells were analyzed 72 h post-transfection for HCV RNA by RT-qPCR. D. Huh7 cells were transfected with pmStrawberry-ATG4BC74A (ATG4B-DN). At 48hr post transfection, immunofluorescence images were taken for ATG4BDN (red) and nucleus (blue). Scale bar, 10μm. E. Infected Huh7 cells (>90% infected) were either mock-transfected (empty plasmid) or transfected with pmStrawberry-ATG4BC74A (ATG4B-DN). Transfected cells were stained for NS3 as described in materials and methods then analyzed by flow cytometry at 72 h post-transfection for NS3 expression gating on red-fluorescent cells (pmStrawberry-ATG4B-DN positive cells). F. Huh7 cells were either transfected with an empty plasmid (mock) or a plasmid encoding a conjugation-defective dominant negative form of ATG5 (ATG5-DN) or ATG12 (ATG12-DN) tagged with HA or Flag respectively. Cell lysates were analyzed by Western blot at 72 h post-transfection for elongation complex formation (ATG5-12/16L1), ATG5-12 conjugation, and LC3-I to LC3-II conversion using anti-ATG5 and anti-LC3, respectively. The expression of ATG5-DN and ATG12-DN was verified using anti-HA and anti-Flag, respectively. β-actin was used as loading control.

The involvement of LC3 in HCV replication-cycle has been addressed previously. LC3 was found to favor HCV translation early in infection with no remaining proviral effect in chronically infected cells [19, 29]. Here, we overexpressed an ATG4B dominant negative protein to evaluate the putative implication of LC3-II in HCV replication-cycle. Indeed, ATG4B is a cysteine protease that prepares LC3-I for conjugation through a proteolytic process essential for LC3-II formation [47]. Overexpression of the ATG4B-DN has been shown to inhibit LC3 lipidation and its downstream-dependent events [48]. Indeed, upon overexpression of ATG4B-DN in Huh7 cells, we observed a 0marked decrease in LC3-II formation and accumulation of P62 which indicates an inhibition of autophagy late events, such as autophagosome maturation and cargo degradation (Fig 10A). In line with our previous report [29], overexpression of ATG4B-DN had no obvious inhibitory effect on HCV polyprotein expression or HCV RNA replication in cells already infected, as indicated by the levels of NS3 and core protein (Fig 10B and 10C). Since these results were obtained upon transient expression using transfection with efficiency around 40–50%, we performed a FACS analysis taking the advantage of the fluorescence property of the mStrawberry tag of ATG4B-DN (Fig 10D) to specifically gate on cells expressing ATG4B-DNin order to assess the level of NS3 expression in this targeted population. The result confirmed that expression of the dominant-negative form of ATG4B has no adverse effect on viral proteins expression (Fig 10E). Together, these results suggest that LC3 lipidation is not mandatory for viral replication in established infection.

### ATG12 conjugation to ATG5 is required for HCV replication in Huh7 cells

Finally, we analysed the importance of conjugation events that precedes LC3II formation by investigating the effect of decoupling the formation of the ATG5-12 conjugate on HCV replication. To achieve this, we overexpressed the dominant-negative forms of ATG5 (ATG5-DN) and ATG12 (ATG12-DN) that have been specifically engineered to impede ATG12 conjugation to ATG5 as previously reported [34]. As expected, the overexpression of the dominant negative forms of these proteins blocked the conjugation of ATG12 to ATG5 as well as its subsequent events, the formation of ATG5-12/16L1 complex, and LC3 lipidation (Fig 10F). Interestingly, when compared to a wild-type ATG12, these conjugation-defective mutants display an adverse effect on HCV lifecycle, as indicated by a decrease in the NS3 and core protein (Fig 10B). Together, these results suggest that the ATG5-12 conjugated form, rather than the individual ATG5 and ATG12 proteins, act as HCV proviral factor.

## Discussion

In a previous study, we showed that HCV RdRp colocalizes and interacts with ATG5, a component of the elongation complex [28]. Here we show that the ATG5-12 conjugate clearly colocalizes on structures that harbor several HCV nonstructural proteins such as NS3, NS4B, NS5A, and NS5B (Figs 2 and 3). We then looked for *in situ* interaction between members of the viral replicase and that of the elongation complex. The results indicate that ATG5 is in close proximity to several HCV nonstructural proteins in infected cells (Fig 4). Since ATG5-12 forms a high molecular weight multimeric complex with ATG16L1 that is absolutely required for autophagosome formation [45], we then analyzed the recruitment of ATG16L1 at the site of HCV replication (Fig 5). Several membranous structures were positive for both ATG16L1 and nonstructural viral proteins. Furthermore, by labeling dsRNA, we were able to show that the replicating HCV RNA colocalizes with the autophagy elongation complex (Fig 7). Together, these results confirm that ATG16L1 is recruited at the site of HCV replication where the elongation complex is formed.

The ATG5-12/16 complex is known to dictate the site of LC3 lipidation [49] due to the E3-like enzyme activity of the ATG5-12 conjugate that is required for the conjugation process of LC3-II which is an essential step in autophagosome formation [30, 50]. Therefore, LC3-II was expected to colocalize with NS4B, NS3, or NS5B. Interestingly we, as well as several other groups [17, 18, 20], were unable to observe colocalization of LC3 with HCV proteins (Fig 8). To further confirm the lack of interconnection between LC3 and HCV replicase component, a PLA experiment that can reveal difficult-to-detect *in situ* interaction was performed (Fig 9). The total absence of interaction or even colocalization was very intriguing since HCV has been shown to trigger the appearance of LC3-II throughout the infected cell [17].

As LC3 was not recruited at the site where the replication complex is localized, we decided to analyze the contribution of LC3-II formation in HCV replication. For this purpose, we used a dominant-negative form of ATG4B (ATG4B-DN) that blocks LC3-II formation. The results demonstrate that although LC3-II formation was indeed severely affected by the ATG4B-DN expression, no inhibitory effect on HCV replication was observed. The results presented in Fig 10 also confirm that the ATG5-12 conjugate is truly important for HCV replication. Indeed, transfection of Huh7 with either ATG5-DN or ATG12-DN, led to a significant decrease in HCV replication. This result not only demonstrates the importance of both proteins but also shows that their conjugation is required for HCV replication.

How exactly the ATG5-12/16L1 complex modulates HCV replication is still unclear. We postulated that HCV infection might trigger *de novo* synthesis of DMV through activation of autophagy since the autophagy elongation complex is essential for proper membranous web formation [29]. Recently, it was shown that DFCP-1, a protein that generates omegasomes, is required for HCV RNA replication. Viral NS5A transiently colocalizes with DFCP-1 on ER protrusions suggesting that omegasomes may provide vesicles on which HCV can replicate [51]. Since the autophagy elongation complex could be recruited at the nascent omegasome for its elongation, it might participate in the creation of the HCV-induced membranous web. Alternatively, the autophagy elongation complex could facilitate membranous web formation through its known capability of enhancing membrane tethering and vesicles aggregation *in vitro* [52].

Previous reports along with the results presented here strongly suggest that the proviral effect of the ATG5-12/16L1 complex is through a noncanonical autophagy process. Indeed, LC3 is clearly not present at the HCV replication site (Fig 8) and is not involved in membranous web formation [29]. Recently a study on another hepatotropic human virus, HBV, demonstrated that the ATG5-12 conjugate but not LC3 acts as a proviral factor [53], suggesting that for some viruses the autophagy machinery rather than the autophagy process have been evolutionary hijacked for their benefits. One possible explanation is that several viruses including HCV have generated strategies to block the antiviral degradative capability of autophagy [21, 54–57]. Indeed, viruses have been shown to block the autophagic flux either by inhibiting autophagosome-lysosome fusion or lysosome acidification. Here, we suggest that by impeding recruitment of LC3 at HCV replication site, degradation of the HCV replication complex by canonical autophagy is eluded. More importantly, a recent study on norovirus, a positive-stranded RNA virus, demonstrated that viral replication complexes can be destroyed via an evolutionary conserved LC3-guided IFN-inducible GTPases antiviral response [32]. Therefore, by preventing LC3 from reaching its replication complexes, HCV may evade this antiviral response as well.

In summary, recruitment of the autophagy elongation complex, which is normally involved in DMV formation, to the HCV replication site, promotes viral replication. Interestingly, the recruitment of the elongation complex is not accompanied by LC3 lipidation at this site. Therefore, HCV infection cycle is more dependent on ATG5-12 conjugation than on LC3 lipidation. Altogether, we believe that HCV has evolved to hijack the autophagy machinery, namely the elongation complex, to promote it replication and to block LC3 recruitment to avoid both antiviral canonical autophagy and LC3-guided INF-inducible GTPases.
